# Autoantibodies to Potassium Channel KIR4.1 in Multiple Sclerosis

**DOI:** 10.3389/fneur.2013.00125

**Published:** 2013-09-02

**Authors:** Raphael Schneider

**Affiliations:** ^1^Department of Medicine, Division of Neurology, University of TorontoToronto, ON, Canada

**Keywords:** multiple sclerosis, autoantibodies, potassium channels, neuromyelitis optica, autoimmunity, plasma exchange

Multiple sclerosis (MS) is an inflammatory demyelinating disease of the central nervous system (CNS); its etiology and pathophysiology remain incompletely understood. Aberrant activation of the immune system is believed to play a central role in MS (1). Despite being labeled an autoimmune condition, MS does not fulfill the criteria of a classic autoimmune disease (2). Myelin-reactive antibodies have been detected in serum and cerebrospinal fluid (CSF) samples of MS patients, their specificity and contribution to the disease process, however, remains a matter of debate. The original concept behind using the B-cell depleting agent Rituximab in MS was based upon the idea that eliminating autoantibodies may improve clinical outcome. While Rituximab reduces disease activity and is associated with a rapid decreases in both B and T cells (3), antibody titers are essentially not affected (4). It was therefore proposed that elimination of B cells may actually hamper cytokine-mediated activation of T cells and results in decreased T cell trafficking across the blood-brain barrier (5). However, modifying the humoral immune response through plasma exchange has shown clinical benefit in certain cases of very active MS (6), suggesting a central role of soluble mediators such as cytokines, antibodies, and complement in MS.

In a recent issue of the *New England Journal of Medicine*, Srivastava and colleagues reported the presence of antibodies to the inward rectifying potassium channel 4.1 (anti-KIR4.1) in MS (7).

The authors observed that purified serum immunoglobulin G (IgG) from MS patients, but not from patients with other neurological diseases, bound to a glial protein in human brain sections. Human brain lysate precipitated with IgG from MS patients revealed KIR4.1 to be the target antigen. Anti-KIR4.1 antibodies were found in serum samples of 47% of MS patients, 1% of patients with other neurologic diseases and in none of the samples from healthy controls. Moreover, anti-KIR4.1 antibodies were detected in the CSF of most MS patients tested. In all MS patients harboring anti-KIR4.1 antibodies, IgG isotypes capable of activating the complement cascade were found. Twenty-four hours after injection of anti-KIR4.1 antibodies and complement into the cisternae magnae of wild-type mice, the authors observed decreased expression of KIR4.1 and glial fibrillary acidic protein (GFAP), a protein expressed by astrocytes.

In their study, Srivastava and colleagues provide data in support of autoantibodies to KIR4.1 as important mediators of inflammation and tissue damage in MS. Their work favors an autoimmune process to be the underlying cause of MS, since specific autoantibodies capable of causing structural damage in normal animals were present in a large number of MS patients. While classical features of MS lesion pathology such as demyelination, axonal loss, and microglial activation were not observed, the authors report evidence for cytotoxicity toward astrocytes.

Until recently, neuromyelitis optica (NMO), an inflammatory disease of the CNS which presents with severe visual failure and extensive transverse myelitis was regarded as a variant of MS. Identification of antibodies against the astrocyte water channel protein aquaporin 4 (AQP4) has led to general acceptance that this condition is distinct from MS (8). The work by Srivastava and colleagues will foster the discussion of how closely MS and NMO are indeed related. Analogous to the pathology described by Srivastava and colleagues, human anti-AQP4 antibodies can induce CNS lesions with loss of AQP4 and GFAP in animals through activation of complement (9). Co-localization of Kir4.1 and AQP4 promotes water permeability in response to fluctuations in osmolarity (10), suggesting a functional link between the two channels. Interestingly, KIR4.1 knock-out mice display impaired myelination of the spinal cord (11), and neurophysiological studies have shown that potassium regulation through Kir4.1 is critical for signal transmission from the retina to the brain (12). Both spinal cord and optic nerve are commonly involved in MS and NMO.

The specific role of anti-KIR4.1 antibodies in the pathogenesis of MS remains to be determined. Does astrocyte dysfunction contribute to the inflammation and demyelinating in MS or could MS even reflect a primary astrocyte disturbance? Is MS indeed part of the growing group of channelopathies, akin to NMO? These are just some of the questions that arise from the identification of anti-KIR4.1 antibodies in MS patients.

The authors found no distinctive clinical characteristics in the cohort of MS patients included in their study. Patients with a clinically isolated syndrome, relapsing-remitting, secondary, and even primary progressive MS tested positive for anti-KIR4.1 antibodies. Whether antibody production correlates with disease activity, or if anti-KIR4.1 antibody titers can help distinguish certain cohorts of MS patients will have to be addressed in future studies. We have learned that accurate distinction between MS and NMO is critical since the two conditions respond differently to certain immunotherapies. NMO can be exacerbated by interferon-beta and severe relapses have been reported in NMO patients treated with fingolimod and natalizumab (13); all three immunomodulators are approved MS treatments. On the other hand, many MS patients do not respond to plasma exchange and it is therefore desirable to identify patients who are likely to benefit from this treatment. It is conceivable that anti-KIR4.1 antibody status will become an important factor to distinguish MS subpopulations and it may even emerge as a tool to guide treatment decisions.

## References

[1] McFarland HF Martin R Multiple sclerosis: a complicated picture of autoimmunity. Nat Immunol (2007) 8:913–9 10.1038/ni1507 17712344

[2] Wootla B Eriguchi M Rodriguez M Is multiple sclerosis an autoimmune disease? Autoimmune Dis (2012) 2012:969657 10.1155/2012/969657 22666554 PMC3361990

[3] Hauser SL Waubant E Arnold DL Vollmer T Antel J Fox RJ B-cell depletion with rituximab in relapsing-remitting multiple sclerosis. N Engl J Med (2008) 358:676–88 10.1056/NEJMoa0706383 18272891

[4] Cross AH Stark JL Lauber J Ramsbottom MJ Lyons JA Rituximab reduces B cells and T cells in cerebrospinal fluid of multiple sclerosis patients. J Neuroimmunol (2006) 180:63–70 10.1016/j.jneuroim.2006.06.029 16904756 PMC1769354

[5] Schneider R Mohebiany AN Ifergan I Beauseigle D Duquette P Prat A B cell-derived IL-15 enhances CD8 T cell cytotoxicity and is increased in multiple sclerosis patients. J Immunol (2011) 187:4119–28 10.4049/jimmunol.1100885 21911607 PMC5052068

[6] Cortese I Chaudhry V So YT Cantor F Cornblath DR Rae-Grant A Evidence-based guideline update: plasmapheresis in neurologic disorders: report of the Therapeutics and Technology Assessment Subcommittee of the American Academy of Neurology. Neurology (2011) 76:294–300 10.1212/WNL.0b013e318207b1f6 21242498 PMC3034395

[7] Srivastava R Aslam M Kalluri SR Schirmer L Buck D Tackenberg B Potassium channel KIR4.1 as an immune target in multiple sclerosis. N Engl J Med (2012) 367:115–23 10.1056/NEJMoa1110740 22784115 PMC5131800

[8] Misu T Fujihara K Kakita A Konno H Nakamura M Watanabe S Loss of aquaporin 4 in lesions of neuromyelitis optica: distinction from multiple sclerosis. Brain (2007) 130:1224–34 10.1093/brain/awm047 17405762

[9] Saadoun S Waters P Bell BA Vincent A Verkman AS Papadopoulos MC Intra-cerebral injection of neuromyelitis optica immunoglobulin G and human complement produces neuromyelitis optica lesions in mice. Brain (2010) 133:349–61 10.1093/brain/awp309 20047900 PMC2822632

[10] Soe R Macaulay N Klaerke DA Modulation of Kir4.1 and Kir4.1-Kir5.1 channels by small changes in cell volume. Neurosci Lett (2009) 457:80–4 10.1016/j.neulet.2009.04.010 19429167

[11] Neusch C Rozengurt N Jacobs RE Lester HA Kofuji P Kir4.1 potassium channel subunit is crucial for oligodendrocyte development and in vivo myelination. J Neurosci (2001) 21(15):5429–38 11466414 10.1523/JNEUROSCI.21-15-05429.2001 PMC6762664

[12] Bay V Butt AM Relationship between glial potassium regulation and axon excitability: a role for glial Kir4.1 channels. Glia (2012) 60:651–60 10.1002/glia.22299 22290828

[13] Barnett MH Sutton I Neuromyelitis optica: not a multiple sclerosis variant. Curr Opin Neurol (2012) 25:215–20 10.1097/WCO.0b013e3283533a3f 22487568

